# Loss of the large conductance calcium-activated potassium channel causes an increase in mitochondrial reactive oxygen species in glioblastoma cells

**DOI:** 10.1007/s00424-023-02833-9

**Published:** 2023-07-04

**Authors:** Bogusz Kulawiak, Monika Żochowska, Piotr Bednarczyk, Andrzej Galuba, David A. Stroud, Adam Szewczyk

**Affiliations:** 1 Laboratory of Intracellular Ion Channels, Nencki Institute of Experimental Biology, Polish Academy of Sciences, 3 Pasteur St, 02-093 Warsaw, Poland; 2 Department of Physics and Biophysics, Institute of Biology, Warsaw University of Life Sciences—SGGW, Warsaw, Poland; 3 Department of Biochemistry and Pharmacology, Bio21 Molecular Science and Biotechnology Institute, University of Melbourne, Parkville, VIC Australia; 4 Murdoch Children’s Research Institute, Royal Children’s Hospital, Melbourne, VIC Australia; 5 Victorian Clinical Genetics Services, Murdoch Children’s Research Institute, Melbourne, VIC Australia

**Keywords:** Mitochondrial potassium channels, Glioblastoma, Mitochondria, Mitochondrial respiration, Reactive oxygen species

## Abstract

Mitochondrial potassium (mitoK) channels play an important role in cellular physiology. These channels are expressed in healthy tissues and cancer cells. Activation of mitoK channels can protect neurons and cardiac tissue against injury induced by ischemia–reperfusion. In cancer cells, inhibition of mitoK channels leads to an increase in mitochondrial reactive oxygen species, which leads to cell death. In glioma cell activity of the mitochondrial, large conductance calcium-activated potassium (mitoBK_Ca_) channel is regulated by the mitochondrial respiratory chain. In our project, we used CRISPR/Cas9 technology in human glioblastoma U-87 MG cells to generate knockout cell lines lacking the α-subunit of the BK_Ca_ channel encoded by the *KCNMA1* gene, which also encodes cardiac mitoBK_Ca_. Mitochondrial patch-clamp experiments showed the absence of an active mitoBK_Ca_ channel in knockout cells. Additionally, the absence of this channel resulted in increased levels of mitochondrial reactive oxygen species. However, analysis of the mitochondrial respiration rate did not show significant changes in oxygen consumption in the cell lines lacking BK_Ca_ channels compared to the wild-type U-87 MG cell line. These observations were reflected in the expression levels of selected mitochondrial genes, organization of the respiratory chain, and mitochondrial morphology, which did not show significant differences between the analyzed cell lines. In conclusion, we show that in U-87 MG cells, the pore-forming subunit of the mitoBK_Ca_ channel is encoded by the *KCNMA1* gene. Additionally, the presence of this channel is important for the regulation of reactive oxygen species levels in mitochondria.

## Introduction

Mitochondrial potassium channels are a group of proteins that enable the rapid flow of potassium ions into the mitochondrial matrix. The primary role of potassium channels in mitochondria is to regulate mitochondrial volume, mitochondrial membrane potential, and the mitochondrial respiration rate [1, 2]. In addition, the activity of mitochondrial potassium channels can regulate the influx of calcium ions into the mitochondrial matrix and affect the synthesis of reactive oxygen species (ROS) by mitochondria [2, 3]. Regulation of these processes may be important for the preservation of mitochondrial function in pathological conditions such as ischemia–reperfusion injury [3–5]. Numerous experiments have shown that activation of potassium channels in mitochondria by potassium channel openers leads to cytoprotection in various tissues [6, 7].

Recently, it has been shown that mitochondrial potassium channels may play an important role in cancer cell function and that channel inhibition may lead to the induction of cell death. Such a phenomenon was observed after inhibition of the mitochondrial Kv1.3 channel, which is overexpressed in various cancer cells [8, 9]. These results suggested that the induction of cell death is caused by an increased level of ROS in the mitochondria due to mitochondrial potassium channel blockage. Similarly, inhibition of the intermediate conductance calcium-activated potassium channel (mitoIK_Ca_) may affect mitochondrial function in cancer cells [10].

One of the best characterized mitoK channels is the large conductance calcium-activated potassium (mitoBK_Ca_) channel [11–14]. This channel has been identified in the inner mitochondrial membrane of many cells and tissues, including the heart, brain, and cancer cells [14–16]. Previous studies have shown the presence of this channel in human glioblastoma, including U-87 MG cells [16–18]. The biophysical properties of the mitoBK_Ca_ channel are very similar to those of the BK_Ca_ channel in the plasma membrane. The channel is regulated by changes in the mitochondrial membrane potential and activated by calcium ions. The mean conductance of the mitoBK_Ca_ channel in U-87 MG cells is approximately 290 pS [17]. The mitochondrial channel is mechanosensitive, activated by the BK_Ca_ opener NS1619, and inhibited by BK_Ca_ channel blockers such as paxilline and iberiotoxin [17, 18]. The activity of the mitoBK_Ca_ channel is also inhibited by heme and hemin and regulated by gasotransmitters such as hydrogen sulfide or carbon monoxide [19, 20]. In addition, mitoBK_Ca_ is a target of various signaling pathways, including phosphorylation or redox signaling [14, 21, 22].

Pharmacological activation of the mitoBK_Ca_ channel in the brain leads to depolarization of the inner mitochondrial membrane and induces an increase in the respiration rate [23]. Moreover, in the model of reverse electron transfer (RET), activation of the mitoBK_Ca_ channel with pharmacological activators leads to a decrease in ROS synthesis by isolated mitochondria [24, 25]. In addition, in U-87 MG cells, the activity of the mitoBK_Ca_ channel is regulated by the electron transport chain (ETC). Reduction of the respiratory chain by administration of substrates (such as succinate) for ETC complexes results in a decrease in potassium channel activity [17]. However, the mechanism of this phenomenon is still unknown.

Despite the unknown mechanism of regulation, interactions between the mitoBK_Ca_ channel and the respiratory chain in U-87 MG cells may occur at the functional and perhaps also structural level [17, 26–28]. It is also not clear whether this phenomenon is general or specific for cancer cells. The above regulatory mechanisms make the mitoBK_Ca_ channel an important element of mitochondrial signaling pathways. The BK_Ca_-type channel is composed of two types of subunits: the α-subunits that make up the pore of the channel and the β-type or γ-type regulatory subunits [29–33]. The α-subunit is encoded by the *KCNMA1* gene, from which multiple mRNA variants can be produced by alternative splicing [31, 33, 34]. Previous studies have indicated that a possible isoform of the α-subunit that may target mitochondria is the VEDEC isoform [31]. This isoform, encoded by *KCNMA1* gene, has been found in cardiomyocytes, and expression of this isoform results in channel activity corresponding to the mitoBK_Ca_ channel [31, 35]. Moreover, deletion of the *KCNMA1* gene in mice resulted in disappearance of 190 pS, most likely mitoBK_Ca_ channel activity, in mouse cardiomyocytes [30]. It should be mentioned, however, that in rat cardiomyocytes this channel has a conductance of about 300 pS [29]. Importantly, the VEDEC isoform is not expressed in U-87 MG cell line [18]. Therefore, other isoforms of this protein may form mitoBK_Ca_ channels. Alternatively, another protein could be responsible for channel activity with similar characteristics in U-87 MG cells.

Therefore, we aimed to verify the hypothesis that the *KCNMA1* gene definitively encodes the protein responsible for the activity of the mitoBK_Ca_ channel in glioblastoma. In this study, we used the CRISPR/Cas9 technique to disrupt expression of the α-subunit of the BK_Ca_ channel in U-87 MG glioblastoma cells. The lack of this subunit was confirmed at the protein level and resulted in a loss of channel activity in mitochondria. We also showed that the removal of the BK_Ca_ channel results in an increase in mitochondrial ROS. However, our data also suggest that the lack of this channel does not affect the organization of respiratory chain complexes or the morphology of mitochondria.

## Materials and methods

### Chemicals

Acrylamid, aminocaproic acid, Bis–Tris, Coomassie blue G–Brillant Blau G250, glycerin, potassium hydroxide, and tricine were obtained from Carl Roth GmbH + Co., Germany. Digitonin and n-dodecyl-ß-D-maltoside were obtained from SERVA, Germany. Ammonium persulfate, potassium chloride, and tris(hydroxymethyl)aminomethane were purchased from BioShop, Canada. PBS–Dulbecco’s phosphate buffered saline w/o magnesium and w/o calcium was from Biowest, France. Protease inhibitor cocktail tablets complete EDTA-free were from Roche, Germany. RNase-free DNase set was from Qiagen AG, Germany. Tween-20 was purchased from Bio-Rad, USA. All other chemicals were from Sigma-Aldrich, USA.

### Cell culture

U-87 MG cells and derived knockout cell lines were grown in Dulbecco’s modified essential medium (Biowest) with 10% FBS (Gibco, USA), GlutaMAX (Gibco), 100 U/ml penicillin, and 100 µg/ml streptomycin (Sigma-Aldrich) at 37 °C in a humidified atmosphere with 5% CO_2_. The cells were split every 3–4 days.

### CRISPR/Cas9

**Fig. 1:**
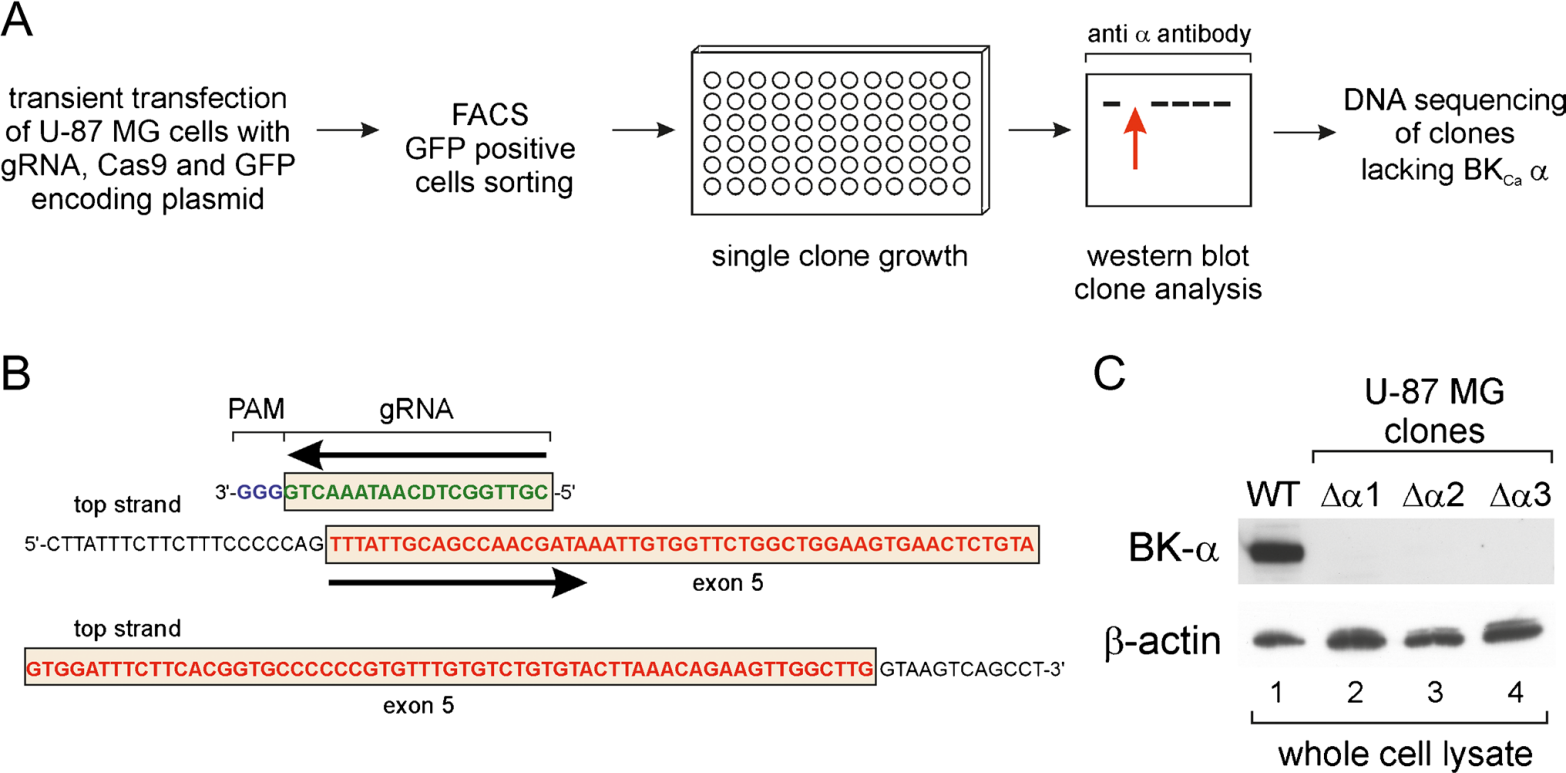
Development of cell lines lacking the BK_Ca_ channel α-subunit. **A** Diagram showing the experimental procedure for the preparation of cell lines lacking the pore-forming α-subunit of the BK_Ca_ channel. **B** Location of the gRNA sequence used in the described project. **C** SDS‒PAGE analysis of cell lysates showing the absence of the α-subunit in newly generated cell lines. Fifty micrograms of protein was used for the analysis. Lane 1, wild-type U-87 MG lysate (WT); lanes 2–4, lysates of U-87 MG clones lacking the α-subunit of the BK_Ca_ channel

Cell lines lacking the α-subunit of the BK_Ca_ channel were developed using CRISPR/Cas9 technology as described in Ran et al. [36]. A schematic drawing showing the preparation of cell lines is presented in Fig. 1A. The gRNA sequence targeting the *KCNMA1* gene (NM_001014797) was selected using the CHOPCHOP web tool (Fig. 1B) [37, 38] and cloned into pSpCas9(BB)-2A-GFP (PX458), which encodes the gRNA, Cas9, and cytosolic localized GFP (Addgene). Cloning of the gRNA sequence into the pSpCas9(BB)-2A-GFP (PX458) plasmid was performed according to Ran et al. [36]. Primer sequences used for gRNA encoding plasmid preparation are presented in Table 1. After transient transfection of wild-type U-87 MG cells, transfected single cells were sorted by fluorescence-activated cell sorting according to GFP fluorescence (Fig. 1A). Cell sorting was performed in the Flow Cytometry Laboratory of the Nencki Institute of Experimental Biology. Single clones were grown in 96-well plates for mutated cell line development. After the appropriate number of cells was obtained, new lines were validated by western blotting to identify knockout clones. The presence of on-target insertions and deletions (indels) was identified by PCR amplification of the targeted genomic region and DNA sequencing (Table 1).

**Table 1:** Primers used in the study for gRNA preparation and sequencing of the disrupted region of the *KCNMA1* gene

Primer	Forward primer	Reverse primer
gRNA preparation	5′-CACCGCGTTGGCTGCAATAAACTGG-3′	5′-AAACCCAGTTTATTGCAGCCAACGC-3′
Sequencing	5′-CTCGCGGATCCTGGCCCTATGAAAACTCAAAAC-3′	5′-CTGCCAAGCTTCACTGCAAATTCTGTCCTTCAG-3′

### MTT assay

Cell growth of newly developed cell lines was assessed by measuring the ability to metabolize 1-(4,5-demethyldiazol-2-yl)-2,5-diphenyltetrazolium bromide (MTT, Sigma-Aldrich). U-87 MG and U-87 MG Δα cells were seeded on 96-well plates at a density of 2 × 10^3^ cells per well in 100 µL of medium. Following the indicated growth time, the medium was replaced with 50 µL of growth medium with MTT (0.5 mg/mL), and the cultures were incubated for another 2–3 h. Then, the medium was replaced with 50 µL of lysis buffer containing 0.4% HCl in isopropanol. The changes in absorbance of formazan dye were measured at 570 nm using a microplate reader (Tecan, Switzerland), with a reference at 655 nm.

### Measurements of mitochondrial respiration

Comparison of mitochondrial respiration of wild-type U-87 MG cells and cells lacking the BK_Ca_ channel was performed using the Seahorse system XFe96 (Agilent Technologies, USA). The Seahorse XF Cell Mito Stress Test (Agilent Technologies) was used in the study. The measurements were performed according to the manufacturer’s recommendations and protocol. Cells were seeded at densities of 2 to 3 × 10^3^ cells per well in 96-well plates provided by the assay manufacturer. The cells were grown in experimental medium (Dulbecco’s modified essential medium with 1 mM glutamine, Agilent Technologies) for 48 h prior to the start of the assay.

The experiment began by measuring oxygen consumption under control conditions, followed by administration of 1 µΜ oligomycin to block ATP synthase activity. Next, 1 µΜ FCCP was administered to measure the maximum respiratory rate. Then, 0.5 µΜ rotenone was injected into the wells to inhibit the activity of mitochondrial complex I. Under all conditions, the measurements were made three times at equal time intervals (according to the manufacturer’s protocol). The results were normalized to the amount of protein in the tested sample. Protein measurement was performed using the Bradford assay (Bio-Rad) at a wavelength of 595 nm with a plate reader (Molecular Devices USA). The analysis of the obtained data was performed using Seahorse Analytics software provided by the manufacturer (Agilent Technologies).

### Mitochondrial isolation

#### Mitochondrial isolation for patch-clamp experiments

Mitochondria were prepared as previously described [17]. For the patch-clamp experiments, U-87 MG and U-87 MG Δα cells were washed and collected in PBS buffer and centrifuged at 800 × g for 8 min. Next, the pellet was resuspended in ice-cold isolation buffer (sucrose 250 mM, HEPES 5 mM, EDTA 1 mM, pH = 7.2) and gently homogenized with a cold Dounce homogenizer. Then, the homogenate was centrifuged at 9200 × g and 4 °C for 10 min. Next, the pellet was resuspended in isolation buffer and centrifuged at 750 × g and 4 °C for 10 min. The supernatant was collected and centrifuged at 9200 × g and 4 °C for 10 min. The pelleted mitochondrial fraction was resuspended in an isotonic solution (150 mM KCl, 10 mM HEPES, pH = 7.2). Each mitochondrial isolation was performed using between 1 and 3 million cells.

#### Mitochondrial isolation for SDS-PAGE and blue native PAGE

Mitochondria for SDS-PAGE and blue native PAGE analysis were prepared using a protocol based on differential centrifugation with introduced modifications [39]. Cells were washed and collected in PBS buffer and centrifuged at 200–500 × g for 10 min. Then, the pellet was resuspended in ice-cold isolation buffer containing 210 mM mannitol, 70 mM sucrose, bovine serum albumin (250 mg per 100 mL of buffer), and 5 mM HEPES–KOH, pH 7.2. Then, for cell membrane permeabilization, digitonin was added to a final concentration of 0.02–0.04%. After 1 min of incubation, the cells were centrifuged at 3000 × g for 5 min at 4 °C. Then, the pellet was resuspended in ice-cold isolation buffer with BSA, homogenized, and centrifuged 2–3 times at 1000 × g for 5 min at 4 °C to remove cell remnants. The supernatant was then collected and centrifuged at 10,000 × g for 60 min at 4 °C. Then, the pellet was resuspended in isolation buffer without BSA and centrifuged at 10,000 × g for 30 min at 4 °C. Then, the pellet was resuspended in isolation buffer without BSA and centrifuged at 10,000 × g for 15 min at 4 °C. This step was repeated once more. The final mitochondrial pellet was resuspended in storage buffer containing 500 mM sucrose and 5 mM HEPES, pH 7.2. Each mitochondrial isolation was performed using between 15 and 30 million cells.

### SDS-PAGE and Western blot

Whole cell lysates and mitochondrial fractions were isolated from U 87-MG and U 87-MG Δα cells as described above. A given amount of sample solubilized in Laemmli buffer (Bio-Rad) was separated by 10% Tris-tricine gel electrophoresis and transferred onto polyvinylidene difluoride (PVDF) membranes (Bio-Rad). After protein transfer, the membranes were blocked with 10% nonfat dry milk solution in Tris-buffered saline with Tween 20 and exposed to an anti-BKα antibody (NeuroMabs, USA, clone L6/60, diluted 1:200), an anti-β-actin antibody (Abcam, UK, 1:1000, no. 8227), anti-SDHA (1:1000, Abcam, no. ab137040), anti-COXIV (1:1000, Cell Signaling, USA, no. 4844), anti-MT-COII (1:1000, Invitrogen, USA, no. A6404), and anti-Core1 (1:1000, Invitrogen, no. 459140). The blots were developed using a secondary anti-rabbit (GE Healthcare, USA, no. NA934, and Thermo Fisher Scientific, USA, no. 31460) or anti-mouse antibody (GE Healthcare, no. NA931) coupled to horseradish peroxidase in conjunction with an enhanced chemiluminescence solution (GE Healthcare). PageRuler Prestained Protein Ladder (Thermo Fisher Scientific) was used to estimate the molecular weight of the analyzed proteins.

### Blue native PAGE

Mitochondria were solubilized in cold blue native buffer pH 7.4 (20 mM Tris, 1 mM EDTA, 50 mM NaCl, 10% glycerol, and 1 mM PMSF) with the following detergents (as marked): 1% digitonin, 1.5% DDM (n-dodecyl-β-D-maltoside), or 1% Triton X-100. The samples were incubated on ice for 15 min and centrifuged at 10,000 × g for 15 min at 4 °C. Supernatants were collected, and loading buffer was added (0.5% Coomassie blue G, 50 mM ε-aminocaproic acid, 10 mM Bis Tris, pH 7.0) [40]. A given amount of sample was separated by 5–10% gradient gel electrophoresis and transferred onto polyvinylidene difluoride (PVDF) membranes (Bio-Rad). After protein transfer, the membranes were blocked with 10% nonfat dry milk solution in Tris-buffered saline with Tween 20 and exposed to antibodies as described above. A High Molecular Weight Calibration Kit for Native Electrophoresis (GE Healthcare) was used to estimate the weight of the obtained protein complexes.

### Electrophysiological single-channel recordings

**Fig. 2:**
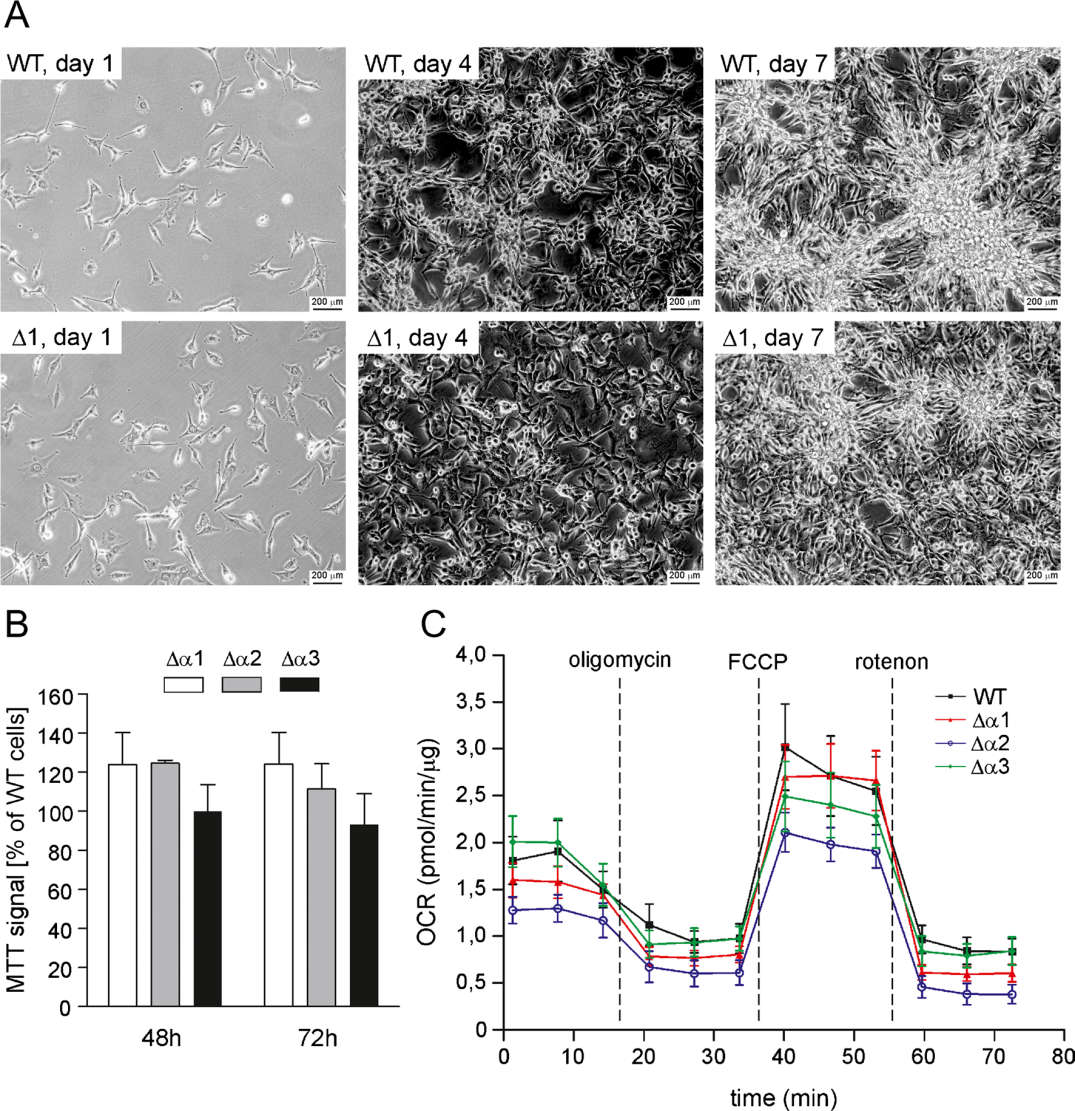
Analysis of growth and oxygen consumption by U-87 MG cells lacking the BK_Ca_ channel. **A** Micrographs of a cell culture of wild-type U-87 MG (WT) cells and the U-87 Δα1 cell line. The pictures show the typical spheres formed by the cells in both cultures (day 7). **B** Cell growth analysis of the newly developed U-87 MG Δα cell lines by the MTT test. The absorbance of metabolized MTT was measured 48 and 72 h after seeding. Cell culture was carried out in 96-well plates. A value of 100% represents the absorbance of the control U-87 MG cells at the indicated time point. **C** Exemplary measurement of oxygen consumption by U-87 MG wild-type cells and cell lines lacking the BK_Ca_ channel. The analysis was carried out using the Seahorse device. The data are presented as pmol/min/µg. The presented values are normalized to the amount of protein in the sample

Patch-clamp experiments using U-87 MG and U-87 MG Δα cell mitoplasts were performed as previously described. In brief, a patch-clamp pipette was filled with an isotonic solution containing 150 mM KCl, 10 mM HEPES, and 100 µΜ CaCl_2_, pH = 7.2. The current–time traces of the experiments were recorded in single-channel mode. A PC-100 puller (Narishige, Japan) was used for pipette preparation. The pipettes were made of borosilicate glass and had a resistance of 10–20 MΩ (Harvard Apparatus GC150-10). The currents were low-pass filtered at 1 kHz and sampled at a frequency of 100 kHz (amplifiers: Axopatch 200B, digidata: Axon 1440A, Molecular Devices). The traces of the experiments were recorded in single-channel mode. Recordings were analyzed using Clampfit 10.7 software (Axon Instruments, Molecular Devices, USA). The conductance of the channel was calculated from the current–voltage relationship (Fig. 2B). Changes in the ion current were determined by event statistics.

### Reverse transcription and quantitative PCR

RNA from U-87 MG wild-type and U-87 MG Δα cells was isolated using the RNeasy Mini Kit (Qiagen AG, Germany) according to the manufacturer’s instructions. Reverse-transcription reactions were performed using a RevertAid First Strand cDNA Synthesis Kit (Thermo Fisher Scientific). The expression levels of selected genes were analyzed using SYBR Select master mix (Applied Biosystems, USA). PCR was performed with a 7900HT Fast real-time PCR system (Applied Biosystems). The primer sequences used in the study are presented in Table 2. The ΔΔCt was calculated to compare the expression of the studied genes in wild-type U-87 MG and U-87 MG Δα cells. The ΔΔCt was defined as follows: ΔΔCt = ∆Ct (U-87 MG Δα) – ∆Ct (wild-type U-87 MG cells). For each of the analyzed genes, the expression level was calculated based on the expression of the *TBP* housekeeping gene (∆Ct). ∆Ct = Ct (gene of interest) – Ct (housekeeping gene). Ct stands for the cycle threshold (Ct) of the tested sample.

**Table 2:** Primers used in the study for quantitative PCRs

Gene symbol	Gene name	Forward primer	Reverse primer
*MT-ATP6*	mitochondrially encoded ATP synthase 6	5′-AATGCCCTAGCCCACTTCTT-3′	5′-TAGGGTGGCGCTTCCAATTA-3′
*MT-CO1*	mitochondrially encoded cytochrome c oxidase I	5′-TTAGCTGACTCGCCACACTC-3′	5′-GGCCACCTACGGTGAAAAGA-3′
*MT-CO3*	mitochondrially encoded cytochrome c oxidase III	5′-CCCTCTCAGCCCTCCTAATG-3′	5′-CATCGCGCCATCATTGGTAT-3′
*MT-ND1*	mitochondrially encoded NADH dehydrogenase 1	5′-ACTACGCAAAGGTCCCAACG-3′	5′-GGCGGGTTTTAGGGGTTCTT-3′
*MT-ND4*	mitochondrially encoded NADH dehydrogenase 4	5′-CTAAAGCCCATGTCGAAGCC-3′	5′-AGGTCTGTTGTCGTAGGCA-3′
*MT-ND6*	mitochondrially encoded NADH dehydrogenase 6	5′-TCAACGCCCATAATCATACAAA-3′	5′-GATGGCTATTGAGGAGTATCCTGAG-3′
*POLG*	DNA polymerase gamma	5′-CAACCCCTAGCTCTGACTGC-3′	5′-CCACGTCGTTGTAAGGTCCA-3′
*POLRMT*	RNA polymerase mitochondrial	5′-GGGACCATCGAAAGGTGTCT-3′	5′-CTTCAGAACAGTGGCCCGAT-3′

The analysis was made from at least 3 independent RNA isolations for each cell line.

### Electron microscopy

Cells were fixed for 30 min with 2% glutaraldehyde in cacodylate buffer, pH 7.2, washed in cacodylate buffer and incubated in 1% osmium tetroxide for 60 min. Next, the cells were dehydrated with a solution containing increasing concentrations of ethanol. Next, the cells were infiltrated with Epon resin. Then, the preparation was cured (polymerized) at an elevated temperature (60–80 °C) for approximately 24 h. The blocks were then trimmed and cut into sections using an ultramicrotome (Leica, Germany). Cutting was performed wet with a diamond knife, and the thickness of the sections was 200 nm. The obtained sections were collected from the water surface directly onto copper-coated microscopic grids with a diameter of 3 mm. The sections prepared in this way were used for observation under a transmission electron microscope.

Image analysis was performed in the ImageJ Fiji program to calculate the following morphological parameters of the mitochondria: organelle circuit, area of a single organelle, Feret diameter (defined as the measure of the greatest distance between two parallel planes bounding the object perpendicular to this direction), and circularity (calculated from the formula *c* = 4π ∗ area/perimeter^2^, where the value 1.0 defined a circle).

### Reactive oxygen species measurements

The level of ROS in the mitochondria of U87-MG wild-type and U87-MG Δα cells was measured using 5 µΜ MitoSOX fluorescent dye (Invitrogen). The whole-cell level of ROS was measured using 7.5 µΜ CM-H_2_DCFDA fluorescent dye (Thermo Fisher Scientific). The cells were seeded on cell culture imaging dishes with a coverslip bottom (Cellvis, Mountain View, USA). After 24–48 h, the cells were incubated in the presence of dye in normal cell culture medium for 30 min at 37 °C and 5% CO_2_ in a humified cell culture incubator. Then, the cells were washed with FluoroBrite™ DMEM (Gibco) with 10% FBS, and confocal images were acquired using an Olympus FV 1200. Image analysis was performed in the ImageJ Fiji program. The values ​​given correspond to the sum of the fluorescence of the area selected for analysis. For standardization of the results, the results are given as percentages relative to the mean fluorescence of the signal measured with wild-type U-87 MG cells. The fluorescence of cells from at least four independent passages was analyzed.

### Statistical analysis

All data are expressed as the means ± SEMs or ± SD from at least three independent experiments. * *p* < 0.05, ** *p* < 0.01, and *** *p* < 0.001 by one-way ANOVA followed by Tukey’s test, 95% confidence interval.

## Results

### Generation of U-87 MG BK_Ca_ knockout cells

U-87 MG cells were used previously to study the regulation of mitoBK_Ca_ channels by the activity of the mitochondrial respiratory chain [17]. However, there was no direct evidence that the *KCNMA1* gene encodes the mitoBK_Ca_ pore-forming subunit of the channel observed with mitochondrial patch-clamping studies. For this reason, we prepared a construct encoding gRNA directing the Cas9 nuclease to the *KCNMA1* gene to disrupt expression and test the hypothesis that a protein it encodes is responsible for the activity of the plasma membrane BK_Ca_ channel and putative mitochondrial isoform [36]. Single-cell clones were screened to identify those where the α-subunit was not present (Fig. 1A) and disruption at the genomic level was confirmed by sequencing (Fig. 1B). We selected three independent cell lines lacking the α-subunit of the BK_Ca_ channel (Fig. 1C).

Because BK_Ca_ inhibition may affect the growth rate of U-87 MG cells [41], we assessed how the lack of BK_Ca_-type channels affects the growth of these cells. We compared the growth rate of our newly developed U-87 MG Δα cell lines with that of wild-type (parental) cells. Comparison of cell culture images suggests that the newly developed cell lines behave in a similar manner to wild-type cells (Fig. 2A). Additionally, we used the MTT assay to quantitatively estimate the growth rate of the tested cell lines. The MTT assay did not reveal significant differences between mutant and wild-type cell lines (Fig. 2B). We found that after 48 and 72 h, growth rate of clones 1 and 2 slightly increased, but clone 3 was comparable to that of wild-type cells. Importantly, these differences while notable were not significant.

### Measurement of mitochondrial respiration

The opening of the mitochondrial potassium channels allows the influx of potassium ions into the mitochondrial matrix, which increases respiratory chain activity unrelated to ATP synthesis by complex V. Therefore, it is reasonable to ask whether the removal of the α-subunit of BK_Ca_ will affect mitochondrial activity related to oxygen consumption. For this purpose, we used the Seahorse XF Cell Mito Stress Test. Figure 2C shows the measurement of respiration of all tested lines in resting conditions after blocking the ATPase (1.5 µΜ oligomycin addition), after maximum uncoupling (1 µΜ FCCP addition), and after blocking the respiratory chain (0.5 µΜ rotenone addition). As shown in the sample measurement, the differences between the lines are not very significant. However, the baseline level of oxygen consumption is low in these cells. One of the clones clearly deviated from the other lines. Our results therefore indicate that BK_Ca_ channel depletion has no major effect on oxygen consumption by the ETC. A summary of the results obtained in this test is shown in Table 3.

**Table 3:** Average oxygen consumption rate measured for the tested cell lines using the Seahorse XF Cell Mito Stress Test

Cell line	Steady state	1.5 µΜ oligomycin	1 µΜ FCCP	0.5 µΜ rotenone
U-87 MG	1.68 ± 0.23	0.95 ± 0.21	2.45 ± 0.33	0.79 ± 0.14
U-87 MG Δα1	1.70 ± 0.23	0.83 ± 0.14	2.54 ± 0.17	0.62 ± 0.05
U-87 MG Δα2	1.30 ± 0.17	0.63 ± 0.15	1.79 ± 0.19	0.46 ± 0.11
U-87 MG Δα3	1.59 ± 0.24	0.79 ± 0.14	2.21 ± 0.23	0.64 ± 0.14

The data are presented as pmol/min/µg ± SD, and values for all measured time points were included. Data were obtained from three independent replicates. Values are normalized to the amount of protein in the sample.

### Patch-clamp analysis of mitochondria isolated from wild-type U-87 MG cells and cells lacking the BK_Ca_ α-subunit

**Fig. 3:**
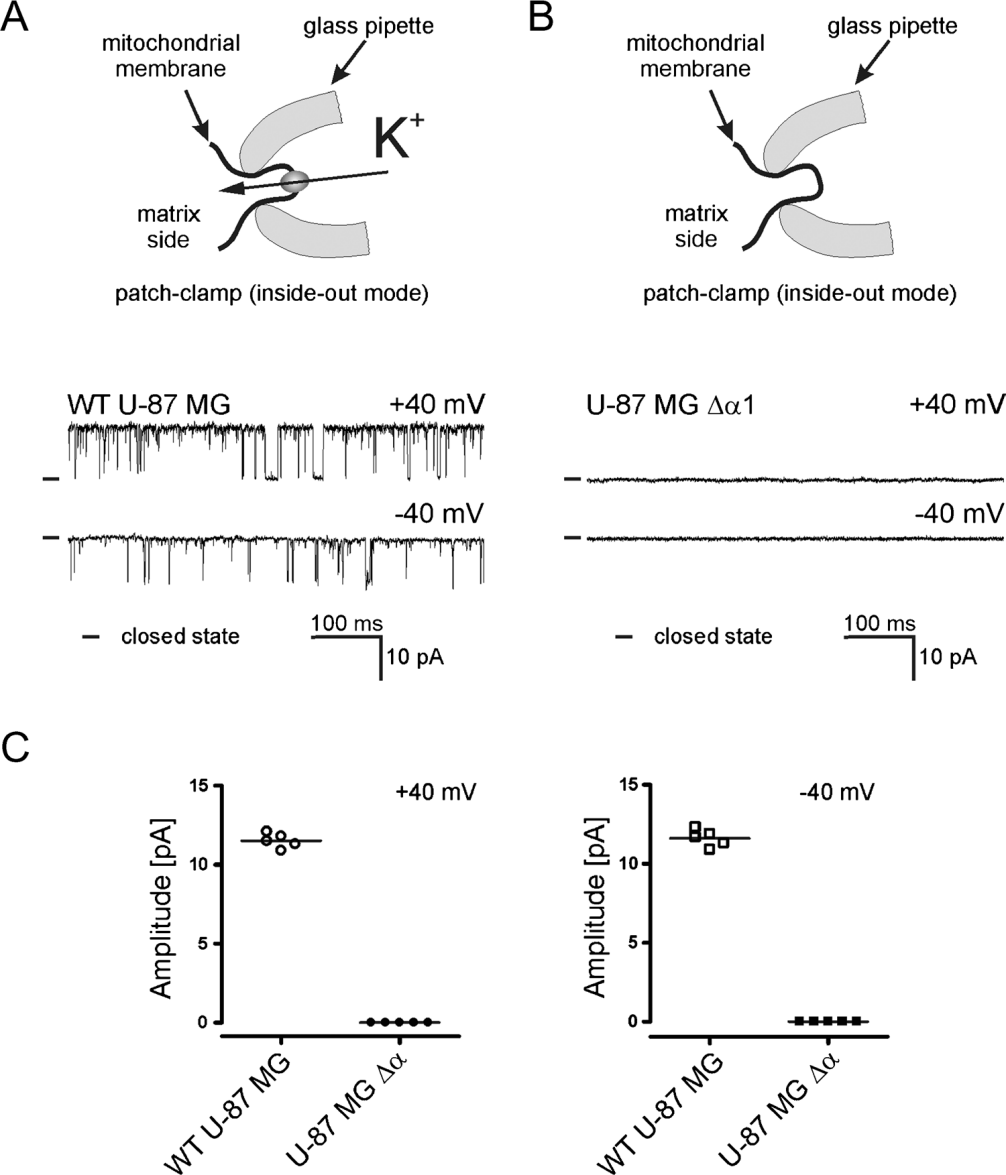
Electrophysiological recording of channel activity in mitoplasts of wild-type U-87 MG cells and cells without BK_Ca_ channels. **A** Schematic depiction of the experimental setup used for the mitoplast patch-clamp recordings (upper panel) and typical recording of the mitoBK_Ca_ channel activity observed in mitoplasts isolated from wild-type U-87 MG cells (lower panel). **B** Schematic depiction of the experimental setup used for the mitoplast patch-clamp recordings (upper panel) and a typical recording observed in mitoplasts isolated from U-87 MG Δα cells (lower panel). **C** Statistical analysis of the ion current amplitude recorded in mitoplasts isolated from wild-type U-87 MG cells and in mitoplasts isolated from cells without the BK_Ca_ channel at + 40 mV (left panel) and − 40 mV (right panel)

Further experiments focused on electrophysiological analysis of the mitoplast isolated from a cell line lacking the BK_Ca_ α-subunit in comparison to a wild-type cell line. In mitoplasts prepared from wild-type U-87 MG cells, we routinely found mitoBK_Ca_ activity (45 activities per 100 patches) (Fig. 3A). The conductance of the channel was close to 290 pS, which is consistent with previously published data [17, 18]. We were unable to detect mitoBK_Ca_ activity in mitoplasts isolated from cells lacking the BK_Ca_ α-subunit (approximately 40 patches) (Fig. 3B). These observations were confirmed by determining the amplitude of the ion current observed in the tested cell lines (Fig. 3C). In addition, it should be noted that no other channel activity was observed in the mitochondria of U-87 MG wild type and U-87 MG Δα cells. These data indicate that the α-subunit is a key protein for mitoBK_Ca_ activity in U-87 MG cells. This observation is important since there has been no unequivocal confirmation that the *KCNMA1* gene encodes the mitoBK_Ca_ channel in glioblastoma cells to date.

### Analysis of the expression of selected genes and the organization of the respiratory chain

**Fig. 4:**
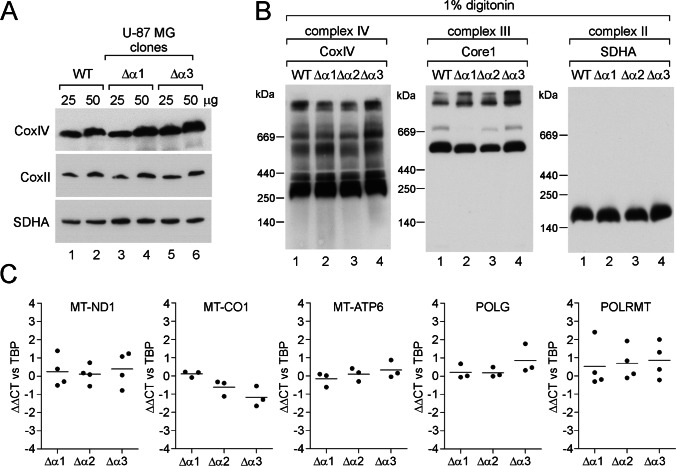
Analysis of the expression of selected mitochondrial proteins and the organization of the respiratory chain in U-87 MG wild-type cells and cells lacking the BK_Ca_ channel. **A** Analysis of the levels of selected ETC proteins using SDS‒PAGE followed by western blotting. Decoration was performed against the CoxII and CoxIV subunits of complex IV and the SDHA subunit of complex II. Crude mitochondria (25 and 50 µg) were loaded on the gel as indicated. **B** Analysis of the organization of the respiratory chain in the tested cell lines using blue native gel electrophoresis followed by western blotting. Fifty micrograms of crude mitochondria was loaded on the gel. **C** Quantitative analysis of the expression levels of selected genes encoded by the mitochondrial genome and genes responsible for the replication and transcription of mitochondrial DNA in the analyzed cells. The reference point was the expression of the marked genes in wild-type U-87 MG cells. The housekeeping gene used in the analysis was TBP

Next, we asked whether loss of the central component of mitoBK_Ca_ affects the abundance of key mitochondrial proteins and the organization of the respiratory chain. Comparison of the levels of several subunits of the respiratory chain in α-subunit knockout clones against wild-type cells did not show clear differences (Fig. 4A). Previous studies have indicated the possibility of an interaction between the respiratory chain and the mitoBK_Ca_ channel [17]. Therefore, we investigated whether the lack of the α-subunit affects the organization of electron transport complexes. The analysis of respiratory chain complexes using the blue native technique did not show differences in the abundance of complexes II, III, or IV (Fig. 4B). Respiratory chain supercomplexes were also largely unaffected. While α-subunit clone 2 lacked the complex III dimer migrating in the 600–700 kDa region, the lack of any reproducible change across all three clones suggests that this is a clone-specific artifact.

Given that the mitoBK_Ca_ channel is thought to be composed of 4 α-subunits with a total mass of approximately 500 kDa, if it was associated with a respiratory complex, its absence would likely result in a large mass shift in the migration of one or more complexes. Therefore, the obtained results do not support the association of the mitoBK_Ca_ with respiratory chain complexes.

The data presented in Fig. 4C show the transcript levels of selected subunits of complex I (*MT-ND1*), complex IV (*MT-CO1*), and ATP synthase (*MT-ATP6*) encoded by mitochondrial DNA as detected by qPCR. In addition, we compared the expression of the mitochondrial DNA polymerase *POLG*, responsible for mitochondrial DNA replication, and a mitochondrial DNA-directed RNA polymerase *POLRMT*, involved in mitochondrial DNA expression. Similarly, no changes in the expression of the above genes were observed. However, fluctuations in the expression level in cells lacking the BK_Ca_ channel are noteworthy.

### The influence of detergents on protein complexes formed by the BK_Ca_ channel in mitochondria

**Fig. 5:**
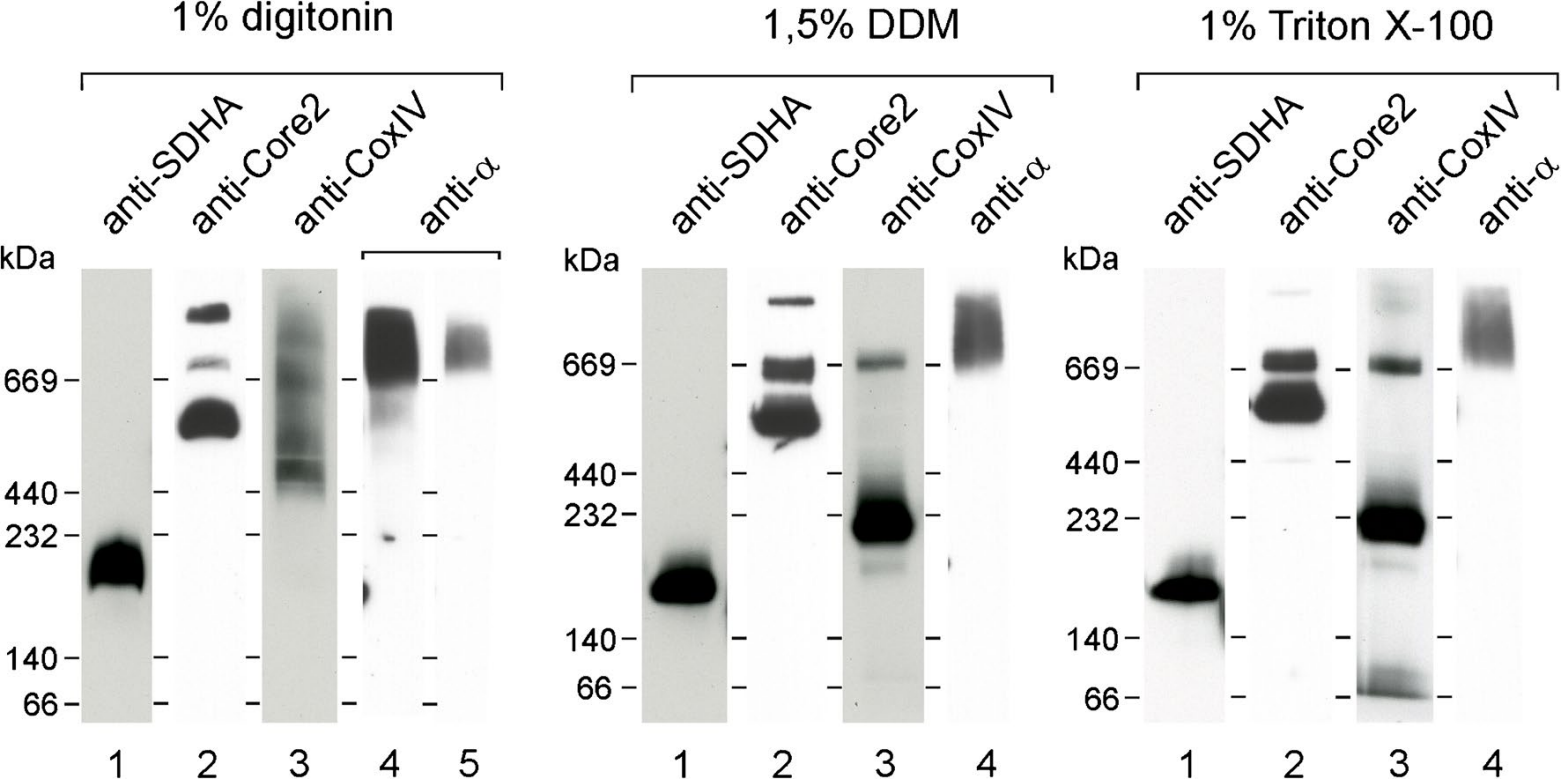
The effect of detergents on the stability of protein complexes in the mitochondria of wild-type U-87 MG cells. Blue native analysis shows staining of respiratory chain complexes and complexes formed by the α-subunit of the BK_Ca_ channel after solubilization with 1% digitonin, 1.5% DDM, and 1% Triton X. Fifty micrograms of protein was applied to the lane for BK_Ca_ channel staining, and 25 µg was applied to the lanes intended for staining respiratory chain complexes

Previous experiments have suggested that BK_Ca_ channel activity in mitochondria is regulated by respiratory chain activity [17]. Moreover, the data indicated that this channel may be structurally related to respiratory chain complexes, in particular, cytochrome *c* oxidase. For this reason, we investigated what complexes are formed by the α-subunit of the BK_Ca_ channel in the crude mitochondrial fraction. Blue native PAGE analysis of the digitonin solubilized mitochondria indicates the existence of high mass complexes (Fig. 5). In parallel, analysis of the respiratory chain complexes III and IV (decoration against core 2 and Cox IV) showed that these complexes do not co-migrate with the α-subunit of the BK_Ca_ channel. Notably, the complexes formed by the BK_Ca_ channel are dispersed and do not form clearly defined bands. A longer exposure revealed a fraction of the signal from the α-subunit that coincides with the signal of complexes III and IV (Fig. 5, digitonin, lane 4). Due to the size of the complex formed by the α-subunit (approximately 500 kDa) and the extended band, the interpretation of this observation is difficult. The use of detergents that affect the stability of complexes and supercomplexes of the respiratory chain, such as DDM and Triton X-100 (Fig. 5), showed that the complexes formed by the α-subunit retain their stability. This result indicates that the potential interaction between the respiratory chain complexes and the mitoBK_Ca_ channel may be dynamic and transient and perhaps not direct.

### Analysis of mitochondrial morphology in wild-type U-87 MG cells and cells lacking the BK_Ca_ channel

**Fig. 6:**
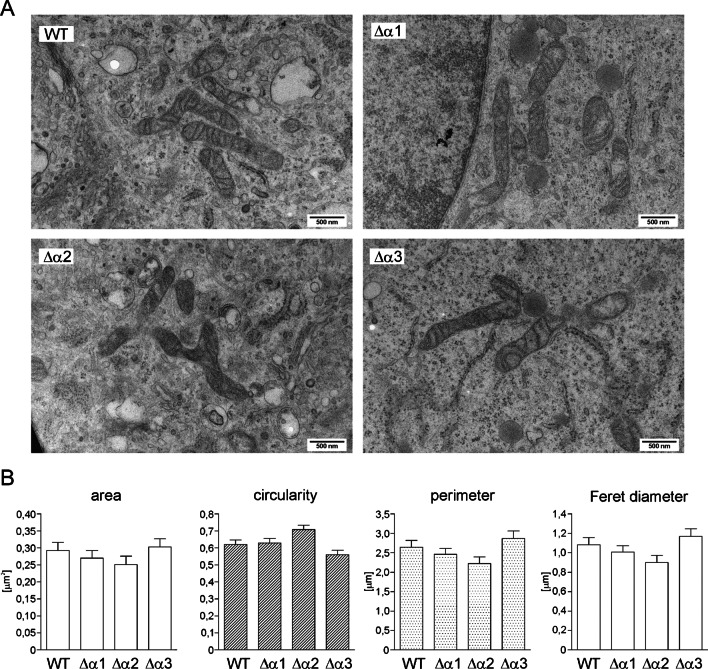
Mitochondrial morphology analysis in wild-type and BK_Ca_-deficient cells by electron microscopy. **A** Sample images of cell interiors obtained with wild-type U-87 MG (WT) cells and U-87 MG Δα cells lacking the BK_Ca_ channel (Δα1, Δα2, Δα3). **B** Quantitative analysis of parameters describing the morphology of mitochondria in the studied cells

The transport of potassium into the matrix is associated with changes in the volume of mitochondria. Therefore, we wanted to determine whether the lack of a channel responsible for the influx of potassium ions would affect the morphology of mitochondria. For this purpose, we used electron microscopy analysis. We analyzed at least 80 mitochondria in all tested cell lines. We considered the area of mitochondria, their perimeter, Feret diameter, and circularity. Sample pictures and analysis of the quantification results are presented in Fig. 6A and 6B. The mean surface area values for wild-type U-87 MG cell mitochondria (WT) were 0.292 µm^2^, while for lines with a deletion of the α-subunit of the BK_Ca_ channel, Δα1 = 0.270 µm^2^, Δα2 = 0.251 µm^2^, and Δα3 = 0.303 µm^2^. The mean perimeter value for wild-type U-87 MG cell mitochondria was 2.641 µm, while Δα1 = 2.456 µm, Δα2 = 2.225 µm, and Δα3 = 2.865 µm. The mean value of the Feret diameter for mitochondria of wild-type cells was 1.08 µm, while Δα1 = 1.006 µm, Δα2 = 0.9 µm, and Δα3 = 1.168 µm. Finally, the mean circularity value for wild-type cell mitochondria was 0.62 (a value of 1.0 represents a circle), while Δα1 = 0.629 µm, Δα2 = 0.708, and Δα3 = 0.560. Mitochondria from U-87 MG Δα2 cells, which also showed the greatest changes in respiration rate, were the most divergent in morphology from wild-type cells. However, the analysis showed no significant differences in the structure of mitochondria in relation to that of the wild-type U-87 MG cells in all studied clones.

### Analysis of the ROS level in wild-type U-87 MG cells and cells lacking the BK_Ca_ channel

**Fig. 7:**
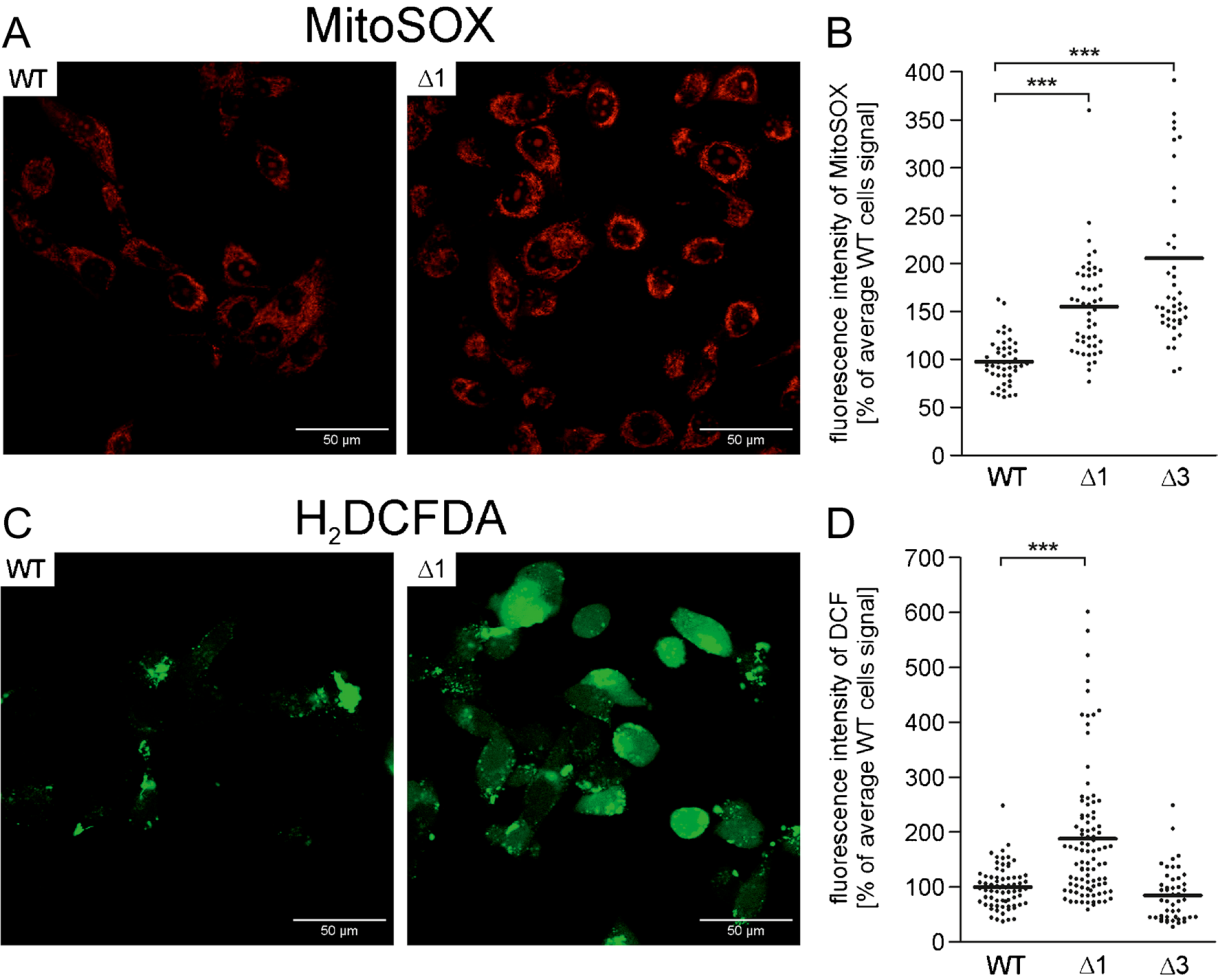
Measurement of the level of reactive oxygen species in mitochondria and whole U-87 MG cells of the wild-type cell line and those lacking the BK_Ca_ channel. **A** Confocal microscopy image of cells stained with the 5-µΜ MitoSOX probe. **B** Quantitative analysis of MitoSOX fluorescence in wild-type U-87 MG cells and cells lacking the α-subunit of the BK_Ca_ channel. **C** Confocal microscopy image of cells stained with the 7.5-µΜ DCF probe. **D** Quantitative analysis of DCF fluorescence in wild-type U-87 MG cells and cells lacking the α-subunit of the BK_Ca_ channel

Subsequent experiments focused on comparing the amount of ROS in mitochondria and whole cells in wild-type U-87 MG cell lines and those lacking the BK_Ca_ channel. To compare the level of ROS in the mitochondria, we used the MitoSOX probe. Under our conditions, the probe was located in the mitochondria, as shown in Fig. 7A. The analysis of the obtained microscopic images showed a higher fluorescence signal in mitochondria in a cell line lacking the BK_Ca_ channel (Fig. 7B). This finding means that ROS levels are elevated in U-87 MG Δα1 and Δα3 mitochondria. A similar observation concerned the monitoring of the level of ROS with the CM-H_2_DCFDA probe. This probe localizes in the cytosol, although in our case, we could also observe fluorescence accumulation in other cellular structures (Fig. 7C). Some of the U-87 MG Δα1 cells showed a higher level of fluorescence, indicating elevated levels of ROS. However, this pattern was different from the pattern observed with the MitoSOX probe. This finding suggests that the probes used monitored ROS in other cellular compartments. However, the average CM-H_2_DCFDA fluorescence in the U-87 MG Δα3 cells was close to that in wild-type U-87 MG cells (Fig. 7D). Taken together, the results indicate an increase in the level of ROS in the mitochondria of cells lacking the BK_Ca_ channel.

## Discussion

The role of the BK_Ca_ channel in glioblastoma, including U-87 MG cells, is being investigated in various aspects, such as its role in cell proliferation or migration. These analyses are part of a wide range of research on the role of potassium channels in cancer cells [42, 43]. The use of the CRISPR/Cas9 technique results in the removal of the BK_Ca_ channel from all cellular compartments, including the plasma membrane and mitochondria. In our work, we focused on the effect of loss of the BK_Ca_ channel on mitochondria.

One of the basic observations in our work is the loss of mitoBK_Ca_ channel activity in the newly developed cell lines. Previous patch-clamp experiments indicated that the observed mitoBK_Ca_ channel in U-87 MG cells is probably similar to the BK_Ca_ channel encoded by the *KCNMA1* gene. This assumption was based on the biophysical and pharmacological properties of the channel, which correspond to a typical BK_Ca_ channel from the plasma membrane [16–18]. However, there was no clear confirmation at the molecular level. This question is important, and the answer is not necessarily obvious. An example of doubts about the molecular identity of the channel is the mitoK_ATP_ channel. Several hypotheses regarding its molecular structure have been proposed regarding this activity [44–46]. In all these cases, the described channel activity showed similar biophysical properties and sensitivity to the same modulators. In U-87 MG cells lacking the α-subunit encoded by the *KCNMA1* gene, we were unable to detect mitoBK_Ca_ channel activity. Therefore, we conclude that the pore of the channel is encoded by the *KCNMA1* gene. However, the question of the α-subunit isoform in U-87 MG mitochondria remains open. Previous studies have indicated that the VEDEC isoform of the α-subunit, encoded by *KCNMA1* gene, can be targeted to cardiac and HEK293T mitochondria and form the mitoBK_Ca_ channel [31, 35]. It was suggested that the specific C-terminus of the protein may be responsible for targeting it to the mitochondria [31]. However, it has previously been shown that U-87 MG cells do not express VEDEC isoform [18]. Due to the complicated splicing process of the α-subunit, which results in the existence of many variants of this protein [33, 34, 47, 48], the answer to this question is difficult. It is known that glioblastoma cells express the BK_Ca_ glioma isoform, so it is possible that it may also target the mitochondria [18, 49]. Interestingly, we did not observe the activity of other ion channels in the inner mitochondrial membrane in U-87 MG wild-type and U-87 MG Δα cells.

Our data indicate that the lack of the α-subunit has no remarkable effect on the expression of mitochondrial proteins, or the organization and function of the respiratory chain in U-87 MG cells. Only one of the three knockout cell lines showed reduced oxygen consumption, suggesting this to be an artifact of the gene editing process. However, previous studies have shown that removal of this channel from cardiomyocytes results in decreases in the activity of ETC complexes [30]. The close functional relationship between the mitoBK_Ca_ channel and ETC also suggested a close structural interaction [17]. Given the size of the mitoBK_Ca_ channel, which is a tetramer of the α-subunit, it is expected that this channel will form complexes close to 500 kDa. The direct, stable interaction between the ETC complexes and mitoBK_Ca_ channel complexes should result in high-mass complexes. Blue native analysis showed that the signal of the α-subunit of the BK_Ca_ channel is diffuse, suggesting the existence of various stable complexes of differing stoichiometry, including BK_Ca_ channel clusters. Perhaps only a small fraction of this channel interacts with ETC complexes, making them difficult to separate and visualize by blue native electrophoresis. Moreover, previous studies have indicated that the mitoBK_Ca_ channel can form functional clusters consisting of multiple channels [35]. Previous studies of the interactions of the BK_Ca_ channel in mitochondria of the brain and heart indicate interactions with subunits of the ETC, ATP synthase, and a number of other mitochondrial proteins, such as the TOM22 subunit of the TOM complex [26, 28]. Our observations may indicate that the ETC and channel complexes in U-87 MG cells may not necessarily form a single structure or complex. Perhaps this interaction is transient or not direct and requires other signaling agents. However, this requires further detailed research.

Despite the lack of a clear effect on the function of the ETC, we observed that the absence of the BK_Ca_ channel in mitochondria results in elevated ROS levels. ROS synthesis in mitochondria may depend on many factors, such as the presence of respiratory substrates or the activity of antioxidant systems [50]. For this reason, identifying a specific mechanism for increasing the amount of ROS in mitochondria is not easy. Previous studies have shown that activation of the mitoBK_Ca_ channel reduces mitochondria RET-induced ROS synthesis [24, 25]. RET may be induced by accumulated succinate, which is observed during reperfusion after ischemia period [51]. In our experimental model, cells were grown in normoxia; therefore, RET is unlikely. However, it is indicated that a change in ROS production does not have to be associated with a significant change in respiration rate. Rather, it is related to a change in the redox state of the respiratory chain [50]. An increase in the reduction of the respiratory chain results in an increase in the amount of ROS produced. The reason for this may be an increase in the amount of respiratory substrates. The BK_Ca_ channel, due to its activity, can affect the mitochondrial membrane potential and respiration rate [1]. The influx of potassium ions into the matrix depolarizes the inner mitochondrial membrane. This induces an increase in the rate of oxygen consumption by respiratory chain but these changes are relatively small [1, 22]. Therefore, the channel may be a regulator of the activity of the respiratory chain and its redox state. The uncoupling activity of the channel allows the respiratory chain to be kept in a more oxidized state when the amount of ROS produced is reduced. It is therefore possible that removal of the channel deprives the mitochondria of this regulatory element. The result may be an increase in the reduction of the respiratory chain, e.g., by increasing the pool of available respiratory substrates [50]. Another option is altered the antioxidant system in the mitochondria [50]. However, this requires a deeper analysis.

It has previously been shown that inhibition of potassium fluxes across the plasma membrane induces cellular ROS elevation, cell cycle arrest, and apoptosis in U-87 MG cells [41]. Elevated levels of ROS in mitochondria after inhibition of mitochondrial potassium channels have been observed in several cancer cell models. Inhibition of the mitoKv1.3 channel results in an increase in ROS, which induces apoptosis in cancer cells such as glioblastoma and melanoma cells [9, 52]. Similarly, inhibition of the mitochondrial IK_Ca_ channel induces an increase in the synthesis of mitochondrial ROS and fragmentation of the mitochondrial network in melanoma, pancreatic ductal adenocarcinoma, and breast cancer cell lines. Loss of the IK_Ca_ channel has also been shown to decrease both glycolysis and ATP production by the mitochondria of breast tumor-derived cells [53]. In glioblastoma cells, inhibition of the IK_Ca_ channel also results in increased sensitivity to ionizing radiation [54]. There are indications that the inhibition of mitochondrial potassium channels may be a promising strategy in cancer therapy, as suggested by in vivo studies [10]. Hence, our observation is consistent with the effects described above.

In addition to the mitochondrial effects, the absence of the BK_Ca_ channel affects the functioning of the entire cell. Previously, knockout of the BK_Ca_ channel using the TALEN technique in osteoblasts resulted in decreased proliferation and inhibited differentiation [55]. Additionally, inhibition of the plasma membrane BK_Ca_ channel affects migration and proliferation and may induce cell death in U-87 MG cells [41, 56]. The effect of the BK_Ca_ channel on U-87 MG cell migration was particularly evident in the stem-like subpopulation of cells where the BK_Ca_ channel is overexpressed [57]. It was also shown that ionizing radiation increased the BK_Ca_ channel open probability, which stimulated the migration and invasiveness of U-87 MG cells [58, 59]. In our studies, removal of the channel did not result in a very significant change in cell proliferation or oxygen consumption (except for one cell line out of three). It is possible that the lack of a channel could be more strongly manifested in specific conditions such as hypoxia or culturing cells in glioblastoma sphere cultures. The average BK_Ca_ current density in U-87 MG cells cultured under hypoxic conditions was significantly higher than that in U-87 MG cells cultured under normoxic conditions [56]. However, our data suggest that oxygen consumption is low in U-87 MG cells. In addition, it must be taken into account that newly developed cell lines probably had to adapt to the lack of the channel. Therefore, the impact of the absence of the BK_Ca_ channel on proliferation or migration should be investigated more systematically in these cells.

In conclusion, our studies showed that the mitoBK_Ca_ channel in glioblastoma U-87 MG cells is composed of pore-forming subunits encoded by the *KCNMA1* gene. The loss of the mitoBK_Ca_ channel results in an increase in the level of reactive oxygen species in the mitochondria. However, the exact mechanism is not clear, as mitochondrial oxygen consumption in cells lacking mitoBK_Ca_ channels did not significantly change. Nevertheless, this effect demonstrates the role of the mitoBK_Ca_ channel in regulating the synthesis of mitochondrial ROS in glioblastoma cells.

## Data Availability

The data that support the findings of this study are available from the corresponding author upon reasonable request.
